# Expert to novice: Understanding the challenges of transition to the specialist community public health nurse - health visitor role and its implications for role retention. A constructivist grounded theory

**DOI:** 10.1016/j.ijnsa.2026.100509

**Published:** 2026-02-13

**Authors:** Lorraine Henshaw, Bill Whitehead, Barry Strickland Hodge

**Affiliations:** a Children and Young Peoples Nursing, School of Health and Society, Room 2:53, Mary Seacole Building, University of Salford, Manchester M5 4WT, United Kingdom; b Scool of Nursing and Allied Health, Birmingham Newman University, Genners Lane, Bartley Green, Birmingham B32 3NT, United Kingdom; c School of Healthcare, Baines Wing, University of Leeds, Leeds LS2 9JT, United Kingdom

**Keywords:** Health visitor, community health nurse, public health nurse, role identity, role retention, role transition, work environment

## Abstract

**Background:**

There are limited studies directly exploring transition to the UK based specialist community public health nurse-health visitor role. This contrasts with abundant evidence exploring transition to a newly qualified nurse which is recognised as difficult and influencing retention to the nursing profession. However, transition to the health visitor role differs fundamentally to transition to the newly qualified nurse as it involves moving from a role where individuals are typically already highly skilled and autonomous practitioners, into a new professional role.

**Aim:**

To develop a substantive theory of the transition to the health visitor role, to support future aspirant health visitors and their educators.

**Method:**

Using constructivist grounded theory, this longitudinal study provides an in-depth understanding of this important transition. It incorporates focus group and interview methods over a series of data collection points, throughout the period of the health visitor course and at 6 months post-completion, with eighteen student/newly qualified health visitors based in a UK university.

**Results:**

This demanding and multifaceted transition is influenced by a range of factors, including role identity, community of practice, individual resilience and the support provided by the wider health visitor team. Data analysis led to the development of a substantive theory incorporating the three core categories of Role Identity, Way of Working and Living the Journey which are illustrated through a conceptual model, providing a visual framework to support this complex transition process.

**Conclusion:**

This research provides a rich evidence-base for the multifaceted and challenging transition to the health visitor role. The greater understanding provided will support future health visitor students, educators and workforce development, potentially enhancing future retention. The findings resonate with similar role transitions, especially those involving a move from expert to novice, extending the relevance outside of the health visitor profession. Future research should include further evaluation of the substantive theory and model with further participants including a wider range of healthcare professional roles, exploration of managing multiple role identities and heightened definition of the health visitor role.

**Contribution of the paper**

What is already known about the topic?

• A robust global evidence base has identified transition to the newly qualified nurse role is a challenging and difficult period that negatively impacts nurse retention.
• There is, in comparison, much less evidence relating to the transition between nursing roles post initial qualification.
• Specific evidence surrounding transition to the specialist community public health nurse (health visitor) role remains anecdotal but does provide awareness of similar difficulties in transition.

What this paper adds

• Provides important new research-based understanding of the complexities of transition to the specialist community public health nurse (health visitor) role.
• Knowledge of the effect of managing multiple identities, changed working practices, the impact of changes and acceptance to community of practice and the importance of protective factors such as resilience and recognition.
• The developed substantive theory provides a conceptual model that helps address transition challenges, guiding discussion and planning for tailored support, which may improve role retention and reduce recruitment needs.

Alt-text: Unlabelled box dummy alt text

## 1 Background

There is a globally recognised body of research which explores transition in a health professional context, most notably within nursing, where the focus is the transition from student to newly qualified nurse. The robust evidence base identifies transition to the newly qualified nurse as a complex period as the newly qualified nurse grapples with the dissonance from their time as a student nurse. Termed ‘transition shock’ by Duchscher (2009), the period is known to negatively influence retention to the profession (Duchscher 2009; Hampton et al., 2021; Ho et al. 2021; Kramer 1974). The evidence base also highlights important factors in a successful transition to the newly qualified nurse (Graf et al. 2020; Murray et al. 2019; Whitehead et al. 2016, Wray et al., 2021), including previous experience, skills acquisition, guidance and acceptance of others and strongly advocates the importance of effective support during this time. Increased awareness and preparation for the newly qualified nurse transition period helps provide appropriate support, which can reduce its effects and promote retention to the profession (Graf et al. 2020; Hampton et al. 2021).

However, despite this important body of evidence highlighting the impact of newly qualified nurse transition, there is, in comparison, much less evidence relating to the transition between nursing roles post initial qualification. This study provides important evidence of the transition to the UK’s post qualification role of specialist community public health nurse -health visitor (please note in the UK the term 'health visitor' is typically used to refer to the specialist community public health nurse- health visitor role; therefore, this paper will now use 'health visitor' throughout). It is derived from anecdotal evidence of the difficulties experienced during transition to the health visitor role (Forbes 2016; Smith 2017), and witnessing first hand, as a health visitor educator, the challenges faced by those undergoing the transition, including withdrawing from the health visitor course and the newly qualified health visitor role. It could be argued that the impact of transition to a health visitor role may be similar to the transition to a newly qualified nurse; however, there are fundamental differences. Essentially, the student health visitor, as a qualified nurse or midwife, is moving from a role as an already established and often highly skilled, autonomous practitioner to a new professional role where they must reconstruct themselves while developing specialist levels of competency and autonomy. Recognising, the complexities associated with the transition to the health visitor role and understanding potential needs during the period, are crucial, as these insights can inform strategies to support and promote a successful transition, potentially enhancing retention to the profession for the future nurses and midwives pursuing a role as a health visitor. This is especially imperative with dramatically reduced health visitor numbers over the last decade, leaving the service under resourced, negatively impacting upon families and children (Morton 2024) and provides further justification for the need for a research-based understanding of transition to the health visitor role.

The health visitor is comparable to other public health nurses across the globe who work as part of communities to promote and protect community health. Likewise, akin to international and national public health nurse colleagues, health visitors are educated beyond initial nurse qualification, undergoing specialist education in public health, normally at post-graduate level. The health visitor role is regulated by the UK Nursing and Midwifery Council, along with other post-registration qualifications (Nursing and Midwifery Council 2024). Over the last decade health visitors have led and delivered the National Healthy Child Programme for 0–5 year-olds, a universally offered health promotion, preventative and support programme in the UK (Morton 2024). The health visitor focus on children under five and their families is similar to Norway’s child focused public health nurse role. Elsewhere, many public health nurses work across the life course.

This research took place at the end of a previous national health visitor recruitment drive (Department of Health 2011a), yet despite recent changes to the education of health visitors, including new standards (Nursing and Midwifery Council 2024), it remains highly relevant. It is important that the findings are shared widely, allowing the knowledge gained to support future recruits as we potentially move towards a second UK national drive to raise health visitor numbers (National Health Service England 2023). The evidence provided by this research also clearly resonates with other similar role transitions, involving a move into specialist and advanced roles or where there is a shift from an expert to novice and therefore has relevance outside of the UK and the health visitor profession.

## 2 The study

This doctoral study was undertaken in an English university commissioned for educating health visitors. Its purpose is to explore how the transition to the health visitor role manifests itself, providing vital knowledge to support future recruits. The transition was explored longitudinally, over time, establishing an in-depth understanding of the process, as it was experienced.

The overall aim of this study was to develop a substantive theory for the role transition from nurse or midwife to health visitor. This will provide an in-depth, evidence-based understanding of transition to the health visitor role and its influencing factors, to support future aspirant health visitors and their educators.

### 2.1 Design and methods

**Fig. 1:**
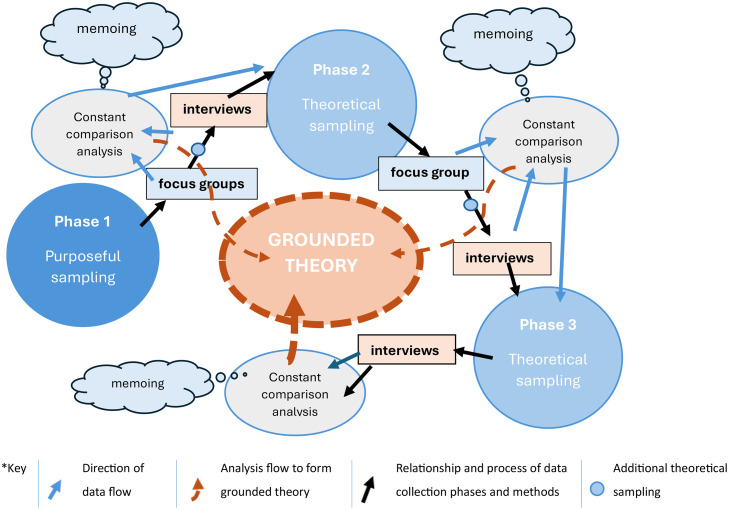
An illustration of the grounded theory process utilised in the study design- adapted from the work of Birks and Mills (2011) p 71.

**Fig. 2:**
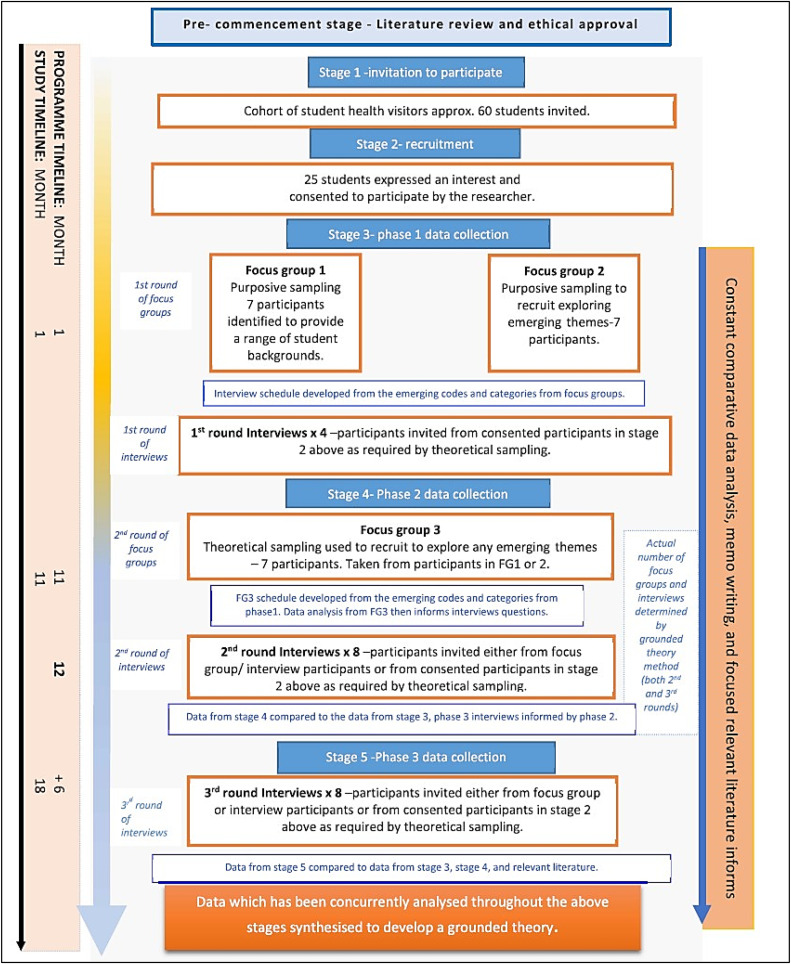
Study design diagram.

The study adopts the constructivist grounded theory methodology (Charmaz 2014), integrating the researcher within the research whilst emphasising the voice of the participants (Birks and Mills 2011). The constructivist grounded theory approach allowed collaborative knowledge creation, enabling the researcher to experience the participants’ world through shared interactions. The key tenets of grounded theory, including theoretical sampling, concurrent data generation, constant comparison analysis, memoing and deep exploration of the data through coding were carefully applied. Fig. 1 illustrates how each of these tenets are incorporated into the study, including identifying where theoretical sampling was employed, the data collection method and chronology of this and each stage of the analysis, alongside the use of memoing. The method of coding is illustrated in Fig. 2.

Mitigation against theoretical oversensitivity was provided through a carefully conducted initial literature review. A more focused review was undertaken during data analysis, where literature surrounding relevant emerging concepts and themes identified during the analysis process, was critically reviewed and incorporated into the analysis, locating the current study within the evidence base and highlighting any original contributions to the field. A flow diagram of the study design can be seen in Fig. 2.

### 2.2 Participants

Participants were recruited at the start of the health visitor programme. Student health visitors were invited to participate through a teaching team member, who although known to the students was independent of the research, lessening any potential power imbalance. Students understood their participation was voluntary, with interested students subsequently contacted by the researcher to provide further information and gain consent. The initial purposive sample (*n* = 14) included students from across all four UK nursing fields and midwifery (nursing in the UK is divided into four distinct fields of practice, each focusing on a specific patient group -see Table 1). Theoretical sampling was then used as needed to achieve data saturation, leading to a final sample of 18. As can be seen in Fig. 2, in stage 2 recruitment, more participants were consented than required to allow for theoretical sampling to occur. No participants withdrew.

**Table 1:** Background demographics of final sample of participants.

Original field of nursing or midwifery	Number	BSc^1^	MSc^2^
Midwife	4	2	2
Child field	2	1	1
Adult field	6	4	2
Mental health field	4	2	2
Learning disability field	2	1	1
TOTAL	18	10 55.5 %	8 44.5 %

1 Bachelor of Science

2 Master of Science

Table 1 presents background demographics, limited to protect anonymity. The sample is split approximately 50:50 between bachelor’s and master’s health visitor programmes. All participants were female, representing the predominantly female health visitor population. Ages ranged from 26 to 55, with 90 % identifying as white British. Time since initial registration varied from 1 to 10 years. The sample aligns with other health visitor student cohorts reported in research (Brook et al. 2019).

### 2.3 Data collection and analysis

Data collection occurred at three intervals over an eighteen-month period. The first and second phases were scheduled as follows: 1) near the start of the programme, approximately one month after commencement and 2) at 10 to 11 months, just before a period of consolidated practice-based learning, when participants were near the end of the programme. The final data collection took place six months after participants completed the programme and qualified as health visitor, at which point they had gained experience in the qualified role.

A combination of focus groups and in-depth interviews were used (Chand 2025). All interviews and focus groups were undertaken in person at the university by the first author. The initial focus group questions were piloted with a group of qualified health visitor colleagues. The focus groups (60–90 min duration) were supported by a co-facilitator/moderator (independent skilled researcher), who took live notes using a flip chart. The notes, used as a form of respondent checking at the end of the focus group, validating the findings, also allowed discussion and co-interpretation with the co-facilitator, to inform reliability (Noble and Smith 2025).

In addition, a series of semi-structured, in-depth and in person, one- to- one interviews were undertaken (Charmaz 2014) (40–60 min duration) providing an individual voice. Initially a pilot interview was undertaken to evaluate the effectiveness of the interview technique. Each of the focus groups and interviews were audio recorded and then transcribed by the researcher. The initial interpretation of the interview transcripts was shared with the participants giving them an opportunity to add anything or to say if they were not happy (Noble and Smith 2025). Data collection and analysis took place in three phases as can be seen in Table 2.

**Table 2:** The timing and relationship between the data collection and analysis phases.

Data Collection Phase	Data Collection Method and Participant	Analysis	Analysis Method	Final Analysis
One (one month into programme)	Focus group x2 (FG^1^1-P^2^1,P6,P11,P12,P14,P16,P18) (FG2-P2,P3,P4,P5,P15,P16,P17). 1 Followed by one-to-one semi- structured interviews with 4 additional participants (P7,P8,P9,P10).	Transcribed and analysed using constant comparison analysis to identify open/initial coding.	Sequential constant comparison analysis over the course of data collection (always prior to next interview or phase).	Develop final categories and grounded theory.
Two (10/11 months into programme)	Focus group x1 (FG-P3,P4,P5,P6,P14,P15,P16). Followed by 8 one-to-one semi structured interviews. (P1,P2,P7,P8,P9,P10,P11,P13).	Transcribed and analysed using constant comparison analysis to identify open/initial coding.		
Three (6 months post qualifying)	8 one-to-one semi structured interviews. (P1,P3,P5,P7,P8,P9,P10,P11)	Transcribed and analysed using constant comparison analysis to identify open/initial coding.		

1 FG (Focus group).

2 P (Participant).

Concurrent data analysis started after the first focus group and continued with each new data collection. Constant comparison analysis was employed whereby newly collected data was compared to existing data, identifying emerging concepts and patterns. Data analysis centred on exploring what has happened/happening, what has changed/changing, potential influences and relevancy to the research aim (Allan 2007). Coding is central to grounded theory analysis. It begins by breaking raw data into labelled segments called codes. These codes are then organized into patterns or categories, forming the basis of an explanatory framework (Charmaz, 2014). The most significant and densely connected categories evolve into subcategories and core categories, which are further analysed and conceptualized to develop the grounded theory (Jones and Alony, 2011).

**Fig. 3:**
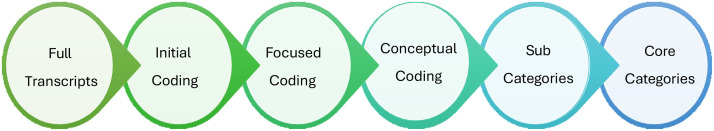
Process of coding within the study.

In this study, initial coding used larger text segments to maintain context and meaning, aligning more closely with participants’ experiences (Charmaz, 2014). Focused coding then refined these initial codes through deeper analysis and synthesis, enabling interpretation of broader sections of data. This iterative process continued until conceptual codes emerged, which were grouped into subcategories. At this stage the identified sub-categories were further conceptualised and grouped into core categories, bringing the fragments of data, split during the initial coding, back together to provide a theoretical explanation of the area of study (Charmaz 2014) (see Fig. 3 for visual explanation).

Data was collected until no new concepts emerged (Birks and Mills 2011). Continued analysis and refining developed three core categories: Role Identity, Way of Working and Living the Journey. Analysis samples and coding were regularly shared with the supervision team.

### 2.4 Ethical approval

Ethical approval was granted by the University of Derby, College of Health and Social Care ethics committee (14–03–14). NHS ethical approval was not required; advice was obtained from the regional NHS Research and Development Department and Clinical Research Network.

Participants were clearly informed of the role of researcher, the study's purpose, and the expectations involved by an independent team member. After expressing an interest, potential participants were provided with detailed information and time for full consideration before providing consent, minimising the impact of power imbalances due to the lead author’s role as a nurse lecturer. Participants were assured that involvement was voluntary and would have no repercussions if declined. Informed consent was obtained at the start and reaffirmed before all focus groups or interviews.

### 2.5 Rigour and reflexivity

The key quality indicators trustworthiness, credibility, dependability, transferability and confirmability (Guba and Lincoln 1985) alongside Charmaz’s (2014) criteria of credibility, originality, resonance and usefulness, were used together to inform this research. These criteria are embedded within the research design (for details please see Table 3). Memoing, employed as a form of filtering, separated out preconceived ideas and beliefs from the data collected through personal reflection and self-critical reflexivity (Charmaz and Thornberg 2021). This was important as the researcher’s existing knowledge as a nurse lecturer may have provided a skewed view of the findings. The memos added to the data for analysis (Birks and Mills 2011), reviewing and revisiting them alongside the participant data allowed ideas to evolve into concrete concepts, facilitated co-production and increased usefulness (Charmaz 2014).

**Table 3:** Methods used to enhance quality and rigour within the study.

Key Criteria	Description
Credibility and dependability	Rich data was collected at each data collection stage. This was subjected to in-depth analysis. Data were digitally recorded and transcribed by the researcher soon after collection to maintain closeness.
Dependability	Data were analysed immediately via initial coding prior to further data collection via focus groups/interviews, enabling theoretical sampling and follow up of emerging lines of inquiry.
Credibility and confirmability	Respondent Checking- Notes were taken during data collection to record key points and used as a form of respondent checking. Previous findings (from earlier data collection points) were also discussed with participants at the start of the next round of data collection, checking and clarifying points to support interpretations (Noble and Smith 2025), Peer Checking- Samples of data analysis and interpretations were shared with supervisors
Credibility, dependability and confirmability.	Triangulation & Saturation**-** Combined data collection methods served to triangulate findings and saturate emerging concepts (Silverman 2013). During the full study data analysis was documented to illustrate coding and categorisation methods and results (see Henshaw, 2021)
Confirmability, Usefulness, Transferability/resonance	A broad range of verbatim quotes have been presented to allow for reader confirmation. Presentations of the findings have constantly prompted feedback regarding resonance in the field of study and more widely in other health care professions.
Dependability and credibility	Care was taken to ensure grounded theory methods throughout the study collection, generation and analysis safeguarding procedural rigour. Presentation of verbatim data demonstrates assessment of ‘fit,’ a quality criterion in Grounded Theory (Birks and Mills 2011)
Originality	Promoted via protecting theoretical oversensitivity and interaction with focused literature (as already discussed) until after initial data collection and analysis. Reflexivity during use of literature employed via memoing (Ramalho et al., 2015). The researcher although a nurse lecturer is not a health visitor and hence in part approaching this research as an outsider researcher or at the very least “an outsider who also has some insider characteristics” (Chammas 2020 p542), reducing the risk of importing predetermined insights and diluting originality. This research offers new insight into field of study
Usefulness, credibility, transferability and confirmability.	Reflexivity -through questioning of potential researcher assumptions, influence decision making and continual self-appraisal within the study (Engward and Davis 2015).
Usefulness, credibility and trustworthiness	Remaining open-minded yet aware of potential preconceptions and influences. Employed self-critical reflexivity and the use of memoing as a form of filtering to identify and separate researcher biases; memos became part of analysis (Charmaz 2014)

## 3 Results

The findings are presented using the three identified core categories. Participant names and focus groups have been replaced below by their identifying code in order preserve their anonymity. For example, the first focus group is FG1, and the eighth participant is P8. The interviews are also identified by their place in the study timeline, for example P1, Int3 is the 3rd round interview for P1. The ‘storyline technique’ (Mills et al. 2014) is also used in this section, enabling and protecting the integrity of the research, whilst providing an engaging and interesting story for the reader. As previously mentioned, the participants represented all fields of nursing and midwifery (see Table 1) providing a range of perspectives.

### 3.1 Role identity

During the early part of the transition to the health visitor role it became evident that the primary identity, as a nurse or midwife, provided a safe place, a “*comfort zone” (FG1)* and encompassed a sense of self (how they saw themselves), articulated by P8 in her declaration, “*I am mental health”*. It also clearly provided a sense of belonging and security, referred to as being “*something I can do without thinking about” (FG3).* Notably for all participants ‘being’ a nurse or midwife appeared particularly important.

*I felt very secure where I was. You know, it's really hard to take that filter off. (P8,Int1)*

The difficulties felt with a move away from this safe place, or comfort zone, was unmistakable with heightened anxiety and stress evident in their transcripts and their behaviour, *“I just cried” (FG1),* “*I broke into tears” (P11,Int3).* The influence of this connection with participants' primary identities was observed throughout the transition, as evidenced by the transcripts from interviews conducted six months after qualification as a health visitor.

*I will be honest if I tell somebody what I do, I’m a nurse. (P3,Int3)*

*It’s the title ‘health visitor’ I don’t like it at all. I think we need to be called health visitor nurses. (FG3)*

Participants expressed concern about the potential erosion of their primary identity (P1, Int3) and articulated a distinct sense of loss. These sentiments were consistently reported across all participants. The challenges associated with student status further intensified feelings of loss and related difficulties.

*I think it’s a bit sad…… a bit of grieving really, you got to leave that …role.* (P8,Int1)

*I don’t think they [other health care professionals] know we are actually qualified nurses. (FG3)*

*I lost a lot of confidence at the start of it- the start of going into practice. You do lose a lot …..it’s a loss of role. (P8,Int2)*

Losing confidence and the need for recognition of their existing qualified status from the wider team and clients was also clearly demonstrated.

*It’s a different area and even though you do feel deskilled as a student I think you do have some valuable points to agree on but you don’t want to say because they’re the experts and you’ll get shot down, you don’t feel valued, it’s a confidence thing. (FG2)*

The process of developing a health visitor identity also involved simultaneously negotiating acceptance into the established health visitor community of practice (Wenger 1998) alongside disconnecting from their previous community of practice. This shift from one community of practice to another is difficult and is also felt as a noticeable loss.

*I did a couple of bank shifts and they were like ‘we miss you’ and that’s the thing I miss sometimes. (P9,Int 1)*

*Culturally health visiting is like a different world and I don’t fit in. I think culturally I just find health visiting very different. (P8,Int3)*

The eventual movement towards a health visitor identity appears both conceptual and behavioural, with immersion in the health visitor practice environment and a period of adjustment and realignment, testing and trialling out the health visitor role seemingly leading to a new way of thinking, as participant 7 put it “*that’s when I think as a health visitor” (P7,Int3)*. Importantly, the primary nurse/midwife identity remains strongly upheld, triggering the need to manage both the primary and health visitor identities.

*I’m a health visitor – well I actually still feel as though I’m a nurse, but I’ll always say I’m a health visitor and a nurse. (P1,Int3)*

### 3.2 Way of working

The way of working changes fundamentally throughout the transition process, which begins with qualified nurses and midwives with existing expertise in their previous roles. However, early into the transition a mismatch between this previous expertise, and that required as a health visitor, culminates in a clear sense of becoming a novice again.

*You’re a student and you think well actually why I have become this? Almost childlike, following a mentor round going what shall I do now? You go into student role and losing initiative as well- it takes over. (P8,Int2)*

*I have felt deskilled at the start- feeling like I was in year one of midwifery and I kept forgetting that I actually have some knowledge and I can use that in my visits- but when I was going out on my visits I was just not using any of it- even the antenatal which I did as a midwife, it was really strange, I think I find it hard to find the boundary to know when to step in and when not to. As a student midwife you’re a student and you don’t know anything, but as a student health visitor you forget that actually you have got a background and you’re qualified. (P10,Int1)*

This also impacts on autonomy, confidence, sense of value and self-worth, leading to a reduced sense of well-being. These changes to levels of autonomy are particularly difficult for many of the participants, as they move from high levels of autonomy in their old role, their comfort zone, to reducing levels of autonomy and self-efficacy as they move through the transition.

*I found it hard because I dropped from a band 7 to a band 5, so I feel like it’s a financial loss but also, I would be that person who was in control. I had a level of autonomy to my job and I think I find it really hard to step back and then be the student. (FG1)*

Changes to the way of working also occurs in terms of managing and delivering care and is another important aspect of the transition. For example, the lack of immediacy of care, and the effect this can have on personal reward systems.

*It was the hardest thing to get my head round …..you can’t say stick a plaster on it and come back in a week- health visiting is not that straightforward. (P5,Int3)*

*It’s sitting with uncertainty- I think that’s a change from nursing- because with nursing you pitch them off, off they go and they’re alright, but sometimes [in health visiting] you might make a difference, or you might not make a difference and sometimes the difference is not immediate – it might be later. (FG3)*

In addition, there is a move to the universalism model of care delivery, which relies, in part, on unsolicited care. Participants really struggled with the notion of feeling unwelcome and selling the service alongside the sometimes negative response to the health visitor from clients and families.

*… I don't do it because of this but when somebody goes “aww, thank you!” and you don't- [in health visiting]! you get chucked out of somebody's house, it’s difficult. (P8,Int3)*

*When I went in as my old self it was a referral to me- ….they wanted you there, whereas now you kind of have to sell the service- you know and I found that difficult in the beginning- I’m actually selling why I am there instead, rather than being wanted. (FG3)*

It was also evident that the participants felt that they should be many health and social care professions at once, articulated as “*all of them, in just one person in” (P8,Int1).* Implying the need to metaphorically wear a different hat, depending on what they found. Clearly, they consider the health visitor role to be more complicated than they imagined, describing it as being an “*amalgamation of other professionals” (P7,Int1).*

*Health visiting – you can’t put that in any little tin and say this is specifically what you do, because you are an advocate, you are a counsellor, whether that be grief, debt, marriage counsellor. You are financial help, you are emotional help, you are everything, you are whatever that family needs you to be at the time. …..As a health visitor, you don’t know what you’re going to face when you knock on that door and whatever it is you do face that’s what you become. You can either become a mental health nurse, you become a general nurse, you become everything. (P11,Int2)*

And yet they appeared unsure about what a health visitor role entails, as they tried to adjust and re-configure themselves, highlighting the confusion and anxiety felt further,

*It felt like a very wide job, it felt linked to too many things and I just couldn’t work out what. (P10,Int1)*

Practise is extremely important in allowing the participants the opportunity to be immersed in the health visitor role, enabling a move to a new way of working. Although to begin with this is overshadowed by the significance of their previous role and existing way of working, this is gradually replaced through a period of adjustment, experimentation, challenging themselves in increasingly complex situations and drawing on their new perspective, realigning and re-configuring themselves, until this became the new normal.

*I think the fact that I have a caseload that I feel responsible for [makes me feel like a health visitor]. It was about a month into qualifying I think because I got a case load that wasn’t really needy, there was nothing you know [challenging] …. I know some newly qualified who have got social care referrals straightway, I didn’t- so I think for that reason it didn’t dawn on me that I am a health visitor. (P7,Int3)*

Others also supported the idea that dealing with the challenges allowed them to now consider themselves as a health visitor and P11 provides a further example:

*I knew I was a health visitor…I think it was when I did one of the most difficult bits, when it was safeguarding….and I said “I’ll do it then”….and I managed to force myself, politely, onto this family. That’s when I thought ‘yeah!!’ Actually! I think it began the process of it. It’s when you get your own case load and you think, yeah I am a health visitor! (P11,Int3)*

### 3.3 Living the journey

The experience of the journey itself is fundamental to the whole transition to becoming a health visitor, it is the way it is experienced, not simply what is experienced. All the participants viewed the transition as a difficult journey, the language used by participants points to the intense nature of this journey.

*I know we're looking for the light at the end of the tunnel, but the journey to get there- it's hard, it's scary and it's a rollercoaster and I don't know about any of you, but I hit rock bottom the other day …because everything just got on top of me, all the work and… I'm starting to get up again now. (FG1)*

*I called my portfolio “the long road.” and it had a hill going up and I only found it by accident, it wasn't that I was looking for a picture for it, it was just there and I thought ‘that is how it is, isn't it?’ (P1,Int3)*

There are many unanticipated threats felt as participants negotiate this vulnerable and challenging time.

*At the start, it was very overwhelming, I couldn’t get to grips with anything. I was finding it very hard …it felt overwhelming and I thought ‘oh god can I get through this year’. (P10,Int1)*

These threats, including losses, are experienced simultaneously and felt very personally,

*…… So, you start on a high, don't you? “Oh, I've got this job and I'm so happy!” I was telling everybody “well I'm going to be a health visitor” like “look at me! I'm so good!“ and then you get into it and then you think “shit! It was so much easier at my last job”. Yeah. I thought, “oh my god, I'm not valued as a team member!” I can't do anything, I'm a nurse with all these skills, I'm losing them-you know? (P1,Int3)*

Interestingly it was clear the relationship participants had with their mentor or practice teacher was very challenging for some and at times their interactions appeared to create a barrier to the transition.

*They keep saying things like, it’s transferable skills but in the other breath they say I need to leave them behind and I can’t. (P8,Int2)*

*You were made to feel like you were a student! Really difficult! I mean you do need to be a student but it's the way that you are treated as a student. I think it's a culture where you're really scrutinised and it makes you feel like that big [made small gesture with thumb and index finger- signifying like nothing]. (P1,Int3)*

Conversely, a supportive network of the established health visitor community was fundamental to alleviate the series of challenges, threats and negotiations which are encountered in many aspects of becoming a health visitor. For example, in the challenge in determining access to a new community of practice, important in the formation of a professional identity (Wenger et al. 2002), the role of community of practice members is crucial as they act as the gatekeepers.

*I didn't really have a great time for the first five months really, but kept going you know, I must have seen the light at the end of the tunnel, really and thought just keep planning ahead, but – and then I've had really good support from my mentor and it's really made a difference. (P7,Int2)*

This support network is also important as a source of recognition in this journey, initially for existing skills and expertise and in providing trust in competencies as the students begin to work in the health visitor role.

*They actually would come and seek me out to come and ask me things and that made me feel really happy, and then other people in the team would involve me in the conversation. (P8,Int2)*

The motivation levels of the participants changed through the transition, starting very positively and dipping as they realise the nuances of the health visitor role and face the many challenges of transition. Motivation and continued aspiration therefore fluctuate up and down throughout the journey and can be positively influenced by several factors. This includes an effective support network and the student’s own levels of determination and tenacity.

*I think it’s very hard work and a very hard job. Without the bigger picture and without the passion would I still be here? (FG3)*

*I find I'm insecure about being an expert to being a novice. I don't know if I'm ready to do that. Somebody said to me, who is a friend who finished the last course. She said it's like having a baby-you forget how hard it is after you've done it…, you're just on that treadmill. Its resilience isn't it [I need] and peer support, professional support. (P8,Int2)*

Sometimes participants felt the challenges were too difficult and they really struggled to move forward into the role, choosing to return to their previous roles.

*Well, I actually want to go back into mental health more now than I did in the beginning. (P9,Int2)*

However, most participants remained positive and enthusiastic and held onto the goal of completing the programme and becoming a health visitor,

*I think I’m probably more passionate. I was passionate to begin with, but my passion’s grown even more. (P11,Int2)*

Resilience was noticeably important and the need to *“get up again” ‘*(FG1) and “*get through”* (P7) were frequently referred to by the participants as they expressed their tenacity and determination.

*I’ve found out I have to find things out for myself and this is how I will start learning. I will ask who else I can but I will have to think outside the box and know that I haven’t got that support but I’m not relying on somebody to help me, I’m doing that for myself. (P1,Int2)*

For most participants, after periods of adjustment, realignment and reconfiguration, nearing the end of the journey they recentred themselves with increased levels of autonomy, confidence and improved wellbeing.

*And then consolidation hits you and you're like “oh! this is how you be a health visitor, actually I can do this!” because then your work's handed in isn't it and you're not doing it every night, come home and you're on the computer. So, you've not got that and you kind of…. you settle into normal life, so your routine and you're back and it's not that bad. (P1,Int3)*

All but two participants, who had significant doubts about the role throughout the transition, were very positive with some already in specialist health visitor roles, taking on additional responsibility and others planning and thinking about their future professional development and where this might take them.

*What we’re talking about is fulfilling potential, if it was going to be easy I wouldn’t have fulfilled my potential, this has broadened my horizons, look, I’m looking into FNP [Family Nurse Partnership]! I never thought I’d ‘be a band six let alone a band 7 but when looking at those opportunities…… I’m thinking oh my god! (P1,Int3)*

P11 epitomises the majority of participants as they reach the end of the study and have moved into the health visitor role,

*It has been like a roller-coaster. The transition of doing something that I knew, and that I was good at, to then go and re-learn everything…it was like starting from scratch……. I loved every minute, even the really hard bits. ……Ask me now if I'd go through it again? Possibly, I don't know. I'd like to think no I wouldn't, but I know I would, I really would. (P11,Int3)*

The journey is undeniably difficult with influences from a wide range of factors including high levels of anxiety and emotion, personal resilience and recognition and support from others. Sadly, for a small minority the journey was not fully completed or sustained as they left the health visitor role to return to their former role (*n* = 2).

## 4 Discussion

The findings of this study demonstrate the transition to the health visitor role is challenging and influenced by a range of factors, including role identity, community of practice, individual resilience and the support provided by the wider health visitor team. Encompassing the process are three core categories of Role Identity, Way of Working and Living the Journey.

The challenge and subsequent reformation of role identity is a key component in the transition process and is influenced by a complexity of factors, including the strong connection felt to the existing nurse/midwife identity. The management of the strong connection felt to the primary identity of nurse/midwife is complicated. The study’s findings demonstrate the challenges, numerous threats and lack of recognition impact the transition process, causing it to falter. The widely used concept of Community of Practice (Lave and Wenger 1991) helps to understand the challenging effects of changing role and hence, identity, as belonging to a community of practice is strongly related to identity formation (Wenger et al. 2002). Ibarra (2005) also makes a strong association between transition and identity, suggesting transition is deeply intertwined with identity transformation which can feel threatening on multiple levels, something that was evident in the participants in the current study. Undoubtedly the salient primary identity and the establishment of the health visitor identity are both important to participants and therefore a form of mutual collaboration between the two identities was needed, allowing the two identities to work together successfully. Caza and Creary (2016) also found that having multiple professional identities is complex and managed by the way individuals identify with, and relate to, the different professional roles, whilst maintaining their sense of self. Although not all, the majority were able to find a way to manage the two identities in a mutually supportive and effective way.

Hughes-Morris and Roberts (2017) explored the issues of returning to the student status in both health visitors and school nurses and although their work relates in a number to ways to this research there are key differences. These being, both health visitor and school nurse participants, a retrospective data collection and a specific and distinct focus on the move to the student role. The similarities revolve predominantly around the difficulty in moving to the student role, the impact on the loss of status this entails, combined with the resulting tensions, offering support for the current study’s findings in this part of the transition (qualified nurse to student). Hughes-Morris and Roberts (2017) concluded the primary identity of the qualified profession must have recognition, but they did not explore in any depth the reason this concept is important. What the current research adds is the further knowledge of the complexities of identity as a key element of the change to the student role within the transition to the health visitor. This is important in considering the impact this could have for nurses, midwives and other health professionals returning to a student role for professional and workforce development requirements.

The struggle over history to establish a clear professional identity for the health visitor role (Baldwin 2012; Machin et al. 2012), and the multi-faceted and broad nature of the role, created further uncertainties during the transition, adding to the complexities. Not only did the participants find it difficult to disconnect from their original primary identity, they were also uncertain about what they needed to become. This uncertainty created difficulties for students to prepare themselves, the ambiguity creating tensions in understanding the distinctiveness of health visitor role (Stansfield 2016). An established and clear professional identity is therefore crucial to enable easier and enhanced transition to the role. This need has been partially addressed through the work of Seaman (2022) whose recent work developed a definition for the professional identity of the health visitor profession. This warrants further debate and exploration.

Changes to the way of working create a need for a period of learning to close the developing skills and knowledge disparity as the transition progresses. The environment impacts significantly on this learning process and the existing health visitor community of practice is again vital in offering support through role modelling, peer support and acceptance. Reflective of Wenger et al. (2002), key gatekeepers, including the established health visitor team and practice educators, were critical in recognising and supporting the use of existing expertise from previous roles, nurturing and appreciating the benefits these wider skills may bring to the health visitor role. Wenger (2010) argued the challenge brought by new members to a community of practice should be seen as an enhancement. Significantly, all participants found recognition of their previous experience problematic. However, this provides an aspect of the transition process where actions can easily be implemented.

The intensity of the journey, the experience of dealing with changes and threats to role identity, changes to their established patterns and ways of delivering care and the loss of autonomy and self-worth, is significant. This detailed knowledge of the nuances of transition are important new knowledge and help to understand the impacts of the transition process.

The findings of this study support previous seminal work around transition, including Bridges (2005) and Van Gennep (1960). However, there are clear differences in the way the participants experienced periods of loss and resulting heightened stress, vulnerability and insecurity, throughout the transition. These are not contained within one period of time but reoccur throughout the whole transition process. In addition, the findings also show the process is not unidirectional or simply linear as suggested by other transition theorists (Kralik et al. 2006), with clear evidence of the many threats experienced by participants causing instability in the transition process.

Resilience appears an important attribute within the transition, allowing for quicker recovery from the complex challenges and many threats experienced. In the professional nursing environment, the ability to be resilient is influenced by various factors, including the stress of demanding workplaces and experiences, feeling valued and cared about, the balance of demands from personal and work lives, and a dissonance arising from the difference between the expectation and realities of practice (Hart et al. 2014). The development and enhancement of resilience is therefore paramount, enabling an improved transition experience and should be prioritised within health visitor educational programmes.

**Fig. 4:**
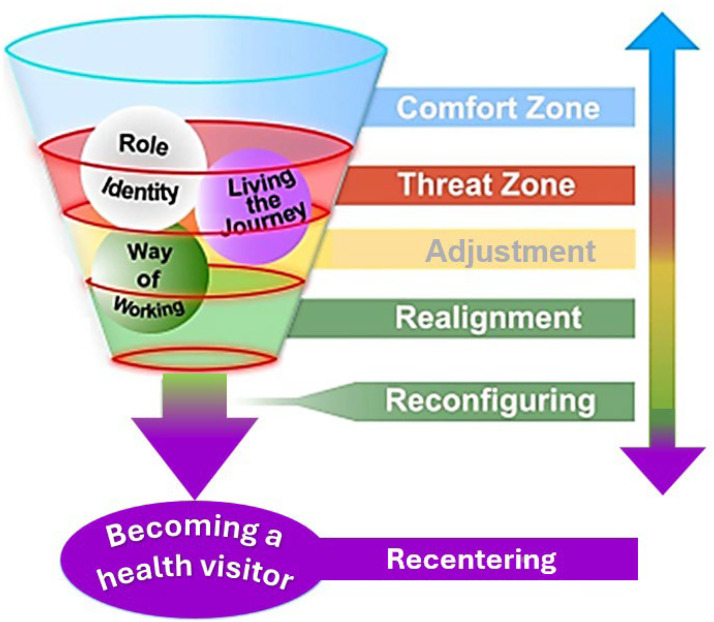
The conceptual model of transition to a health visitor role (Henshaw, 2021, p145).

To illustrate the findings of this study and diagrammatically represent the complexities of the transition to a health visitor role, a conceptual model was generated encompassing the three core categories (Fig. 4). The interrelationships between the core categories are intricate, impacting each other. For example, to allow a transition into a health visitor role, participants each experience simultaneously a new way of working and challenges to identity and both of these are also connected directly to the way the transition journey is experienced. Analysis revealed the three core categories each include progressive stages that participants must navigate to reach the final re-centering stage and fully assume the health visitor role. The conceptual model represents these stages as coloured rings within a cone-shaped funnel, indicating the intended direction of transition. Each category is depicted as a sphere, and movement through the funnel is multidirectional; participants may progress or regress at any point.

The zones are color-coded: **blue** for the comfort zone (starting point), **red** for the threat zone (major challenges), **amber and green** for adjustment, realignment and reconfiguration, and **purple** for re-centering at completion. Importantly, the threat zone, although most intense early in the process, persists throughout the transition and can re-emerge, disrupting progress (signified by red rings). This model provides a useful resource as an explanatory visual framework to support aspirant health visitors, the wider health visitor community and educators, enabling further exploration and consideration of strategies to facilitate the complex transition.

### 4.1 Recommendations

Sharing the findings and conceptual model widely with key stakeholders is recommended, particularly with new and prospective health visitors and those supporting them, including educators. Within student health visitor recruitment, and subsequent education, sufficient regard should be given to the challenges of transition to foster preparedness, personal strategies and peer support, including considering challenges to identity. Raising awareness of the wider health visitor community and their influences during this transition, especially on recognising individual skills and expertise, is vital and should be incorporated into staff development/training opportunities. Enabling resilience via structured support should be a priority in all health visitor educational programmes and practice settings.

Further research should be undertaken to evaluate the theory and conceptual model with additional participants, including with a more diverse range of health care disciplines, providing empirical validation. Additional research is also needed to further clarify the professional identity of the health visitor and to continue to explore the management of multiple professional identities.

### 4.2 Strengths

The conceptual model has already shown value beyond its original context with initial validation provided via feedback from sharing the model more widely, both locally and at national and international conferences. There is important resonance with other role transitions (e.g., advanced clinical practitioner role), and similar transitions to advanced and specialist roles, not just within nursing. The model has been shared with additional student health visitors, qualified health visitors, school nurses, advanced clinical practitioners (including trainees), ultrasonography students and specialist nursing roles, including nurse educators. Feedback was very positive and supports the model of transition’s usefulness beyond the substantive area and its potential national and international relevance.

## 5 Limitations

Researcher familiarity with the field as a nurse lecturer may have introduced bias, potentially influencing both data interpretation and participant responses, despite facilitating contextual insights, participant trust and the careful use of reflexivity and memo writing by the researcher. The limited number of participants, while demographically similar to other studies with student health visitors (Brook et al. 2019), could reduce the transferability of the findings. Additionally, the resulting substantive theory applies specifically to health visiting and has not yet been empirically tested beyond this study.

## 6 Conclusion

This study offers an in-depth exploration of transitioning into the health visitor role, capturing experiences directly from individuals undergoing the transition, as it happened. It therefore, provides an in-depth understanding of the idiosyncrasies of the transition process and has led to the development of a substantive grounded theory, illustrated by the conceptual model. This understanding will help future health visitors and other stakeholders anticipate and address the challenges of this transition.

The core categories of Role Identity, Way of Working and Living the Journey encompass the transition to the health visitor role. It is a challenging, multifactorial and multidimensional, process. Yet with appropriate support, individual determination and resilience, these complexities are surmountable, and this is a largely successful transition. Key aspects of the transition need acknowledgment to minimise the effects of its complexities, including the importance of gatekeepers, and to improve the transition process through effective strategies and support.

The rich evidence provided by this research addresses a deficit in the knowledge base and much needed understanding of transition to a health visitor role. This knowledge helps to recognise and address the student health visitors’ needs during this complex transition. It can potentially reduce attrition and enhance retention in the workforce, improving the experience from all perspectives, including students, the established health visitor community, workforce development teams and educators. Ultimately, it could also encourage lasting health visitor careers.

## Funding statement

This research received no specific grant from any funding agency in the public, commercial, or not-for-profit sectors.

## CRediT authorship contribution statement

**Lorraine Henshaw:** Writing – review & editing, Writing – original draft, Project administration, Methodology, Investigation, Formal analysis, Data curation, Conceptualization. **Bill Whitehead:** Writing – review & editing, Writing – original draft, Validation, Supervision. **Barry Strickland Hodge:** Writing – review & editing, Writing – original draft, Validation, Supervision.

## Declaration of competing interest

The authors declare that they have no known competing financial interests or personal relationships that could have appeared to influence the work reported in this paper.

## Data Availability

The data that support the findings of this study are available upon reasonable request from the corresponding author. The data are not publicly available due to privacy or ethical restrictions.
